# Rat Hepatitis E Virus as Cause of Persistent Hepatitis after Liver Transplant

**DOI:** 10.3201/eid2412.180937

**Published:** 2018-12

**Authors:** Siddharth Sridhar, Cyril C.Y. Yip, Shusheng Wu, Jianpiao Cai, Anna Jin-Xia Zhang, Kit-Hang Leung, Tom W.H. Chung, Jasper F.W. Chan, Wan-Mui Chan, Jade L.L. Teng, Rex K.H. Au-Yeung, Vincent C.C. Cheng, Honglin Chen, Susanna K.P. Lau, Patrick C.Y. Woo, Ning-Shao Xia, Chung-Mau Lo, Kwok-Yung Yuen

**Affiliations:** The University of Hong Kong, Hong Kong, China (S. Sridhar, C.C.Y. Yip, S. Wu, J. Cai, A.J.-X. Zhang, K.-H. Leung, T.W.H. Chung, J.F.W. Chan, W.-M. Chan, J.L.L. Teng, R.K.H. Au-Yeung, V.C.C. Cheng, H. Chen, S.K.P. Lau, P.C.Y. Woo, C.-M. Lo, K.-Y. Yuen);; The University of Hong Kong–Shenzhen Hospital, Shenzhen, China (J.F.W. Chan, C.-M. Lo, K.-Y. Yuen);; Xiamen University, Xiamen, China (N.-S. Xia)

**Keywords:** viruses, zoonoses, immunocompromised, rodents, rat hepatitis E virus, chronic hepatitis, liver transplant, Hong Kong, hepatitis, hepatitis E, HEV

## Abstract

All hepatitis E virus (HEV) variants reported to infect humans belong to the species *Orthohepevirus* *A* (HEV-A). The zoonotic potential of the species *Orthohepevirus* *C* (HEV-C), which circulates in rats and is highly divergent from HEV-A, is unknown. We report a liver transplant recipient with hepatitis caused by HEV-C infection. We detected HEV-C RNA in multiple clinical samples and HEV-C antigen in the liver. The complete genome of the HEV-C isolate had 93.7% nt similarity to an HEV-C strain from Vietnam. The patient had preexisting HEV antibodies, which were not protective against HEV-C infection. Ribavirin was an effective treatment, resulting in resolution of hepatitis and clearance of HEV-C viremia. Testing for this zoonotic virus should be performed for immunocompromised and immunocompetent patients with unexplained hepatitis because routine hepatitis E diagnostic tests may miss HEV-C infection. HEV-C is also a potential threat to the blood product supply.

Hepatitis E virus (HEV) infects 20 million humans worldwide annually (*1*). HEV-infected persons usually have self-limiting acute hepatitis. However, persistent hepatitis can occur in HEV-infected immunocompromised patients who acquire infection by eating undercooked pork, rabbit, deer, camel, or boar meat (*2*–*6*). HEV transmission through blood product transfusion also has been described (*7*).

The diverse *Hepeviridae* family, which incorporates all HEV variants, includes members whose primary host species are terrestrial mammals (genus *Orthohepevirus*) and fish (genus *Piscihepevirus*) (*8*). The *Orthohepevirus* genus is classified into 4 species; HEV variants that have been reported to infect humans belong to *Orthohepevirus* *A* (HEV-A). Five genotypes within HEV-A (HEV-1–4 and -7) cause hepatitis in humans, and 3 genotypes (HEV-3, -4, and -7) can cause chronic hepatitis in immunocompromised patients after foodborne zoonotic transmission (*2*,*6*,*9*,*10*).

In addition to HEV-A, the *Orthohepevirus* genus includes 3 other species: *Orthohepevirus B* circulates in chickens, *Orthohepevirus* *C* (HEV-C) in rats and ferrets, and *Orthohepevirus D* in bats. HEV-C, also known as rat hepatitis E virus, shares only 50%–60% nt identity with HEV-A (*8*). The zoonotic potential of HEV-C is unknown; cases of clinical infection have not been reported. The substantial phylogenetic divergence between HEV-A and HEV-C, especially in critical receptor binding domains, forms a theoretical species barrier (*11*). Serologic and molecular tests for HEV are designed primarily to detect HEV-A, and they might miss HEV-C infections. Therefore, the threat to human health, including blood and organ supply safety, from HEV-C is unknown. We aimed to prove definitively that HEV-C can infect humans and describe the clinical, epidemiologic, genomic, and serologic features of this new zoonosis.

## Materials and Methods

### Study Population

We conducted this study in Queen Mary Hospital, a 1,700-bed tertiary care hospital in Hong Kong. We assessed 518 solid-organ transplant recipients (kidney, liver, lung, and heart transplant) who were followed up in Queen Mary Hospital for persistent biochemical hepatitis from January 1, 2014, or date of transplant (whichever date was later) through December 31, 2017. We defined persistent hepatitis as elevation of alanine aminotransferase (ALT) >1.5 times the upper limit of the reference level for a continuous period of >6 weeks. For patients whose ALT met this definition, we reviewed clinical records, ultrasonogram results, endoscopic retrograde cholangiopancreatography results, and laboratory results to identify the likely cause of hepatitis. We considered patients to have hepatitis B virus (HBV), hepatitis C virus (HCV), or cytomegalovirus (CMV) reactivation if any of these viruses were detected in blood during the hepatitis episode. In patients with no identifiable cause of hepatitis, HEV IgM ELISA screening was performed, in accordance with the usual practice in Queen Mary Hospital. HEV infection was diagnosed if the HEV IgM assay was positive, and persistent HEV infection was diagnosed if HEV viremia in patient plasma lasted for >3 months. PCR sequencing was performed for speciation of HEV isolate. We obtained ethics approval from the Institutional Review Board of the University of Hong Kong/Hospital Authority West Cluster. We obtained written informed consent from all patients with persistent HEV infection.

### Nucleic Acid Detection for Hepatitis Viruses and HEV Complete Genome Sequencing

We designed 3 in-house–developed reverse transcription PCRs (RT-PCRs) to detect HEV (Technical Appendix Table 1). Hepatitis A virus (HAV) RNA and CMV DNA detections were performed using in-house nucleic acid amplification tests. HBV and HCV viral loads were quantified using commercial kits (COBAS TaqMan, Roche, Basel, Switzerland; and RealTime HCV, Abbott, Chicago, IL, USA, respectively).

We sequenced the PCR product of the pan-*Orthohepevirus* RT-PCR using the RT-PCR primers. Because the RNA-dependent RNA polymerase sequences of patient HEV isolates clustered with rat HEV-C strains, primers for complete genome amplification were designed by multiple alignment of rat HEV-C genomes in GenBank (Technical Appendix Table 2). We used these primers for complete genome sequencing of HEV in patient feces (strain LCK-3110). We constructed phylogenetic trees using MEGA6 with the general time reversible plus gamma model (*12*).

### Cloning and Purification of Recombinant HEV-A and HEV-C Open Reading Frame 2 Protein

We used specific primers (online Technical Appendix) to amplify the genes encoding the 239 aa immunogenic recombinant peptides of HEV-A (genotype 4) and HEV-C. Cloning the amplified genes into a bacterial expression vector, expression in *Escherichia coli*, and protein purification were performed as previously described (*13*,*14*).

### Antibodies Against HEV-A and HEV-C

Polyclonal antibodies against the HEV-C recombinant protein were raised in mice (online Technical Appendix). In addition, we used 2 murine monoclonal antibodies (mAbs) against open reading frame (ORF) 2 antigen of HEV-A in this study.

### Serologic Testing

We conducted HEV antibody screening for patients with unexplained persistent hepatitis using HEV IgM and HEV IgG commercial ELISA kits (Wantai, Beijing, China) and detected hepatitis B surface antigen (HBsAg) using the ARCHITECT HBsAg chemiluminescent microparticle immunoassay (Abbott). HAV IgM and HCV antibodies were tested using VIDAS immunoassay kits (bioMérieux, Marcy-L'Étoile, France). For investigation of the HEV-C transmission event, we subjected patient and donor serum to HEV-A and HEV-C Western blots using polyclonal antiserum from mice inoculated with HEV-C protein and mAbs as controls. ELISAs using recombinant HEV-A and HEV-C protein-coated plates were designed based on the method described by Shimizu et al. with modifications (*15*). We set cutoffs and interpreted results to differentiate HEV-A– and HEV-C–specific serologic responses (online Technical Appendix).

### Virus Culture

We selected cell lines A549 (lung adenocarcinoma), Huh-7 (hepatocellular carcinoma), and Caco-2 (colorectal adenocarcinoma) to investigate whether human cell lines could support HEV-C growth. Cell lines were chosen if they supported growth of patient-derived HEV isolates or HEV infectious clones (*16*–*18*) (Technical Appendix). We subjected supernatants and lysates to HEV-C quantitative RT-PCR (qRT-PCR) and immunostaining.

### Immunohistochemical and Immunofluorescence Staining

We conducted immunohistochemical staining of formalin-fixed paraffin-embedded liver tissue sections and infected A549 cell culture monolayers using HEV-C polyclonal serum antibodies and HEV-A mAbs. We performed immunofluorescence staining of permeabilized infected cells using HEV-C polyclonal antiserum (Technical Appendix).

### Epidemiologic and Environmental Investigation

We retrieved organ and blood donor serum for HEV ELISA, Western blot, and HEV-C qRT-PCR. To survey density of rat fecal contamination and collect environmental specimens for HEV-C qRT-PCR, we visited the patient’s housing estate on November 22, 2017. Furthermore, from deep freezers we retrieved archived *Rattus* sp. liver, spleen, rectal swab, and kidney specimens collected during 2012–2017 within a 2.5-km radius around the patient’s residence for preexisting pathogen surveillance programs and subjected them to HEV-C qRT-PCR. The HEV-C ORF2 fragment of qRT-PCR–positive specimens was sequenced using additional primers (Technical Appendix Table 3).

## Results

### Hepatitis E Incidence in Transplant Recipient Cohort

Of 518 patients, 52 (10.2%) had persistent hepatitis (Table 1). Five (9.6%) patients with hepatitis tested positive for HEV IgM; 4 of these were kidney transplant recipients, and 1 was a liver transplant recipient. Together with reactivation of chronic HBV infection, HEV was the third most common cause of viral hepatitis in the local transplant population. Of the 5 patients, plasma HEV-A qRT-PCR of 3 renal transplant recipients was positive; another renal transplant recipient tested negative for HEV RNA. We have previously reported the clinical details of the 3 HEV-A–infected patients (*9*). Rat-derived HEV-C infection was diagnosed in the liver transplant recipient, which accounted for 1.9% (1/52) of persistent hepatitis in our cohort.

**Table 1:** Demographic and clinical characteristics of solid organ transplant recipients, Queen Mary Hospital, Hong Kong, January 1, 2014–December 31, 2017*

Characteristic	Result†
No. transplant recipients	518
Organ transplanted	
Kidney	430 (83.0)
Liver	61 (11.7)
Heart	16 (3.1)
Lung	10 (1.9)
Combined kidney and liver	1 (0.2)
Median age, y	56
Sex	
F	203 (39.2)
M	315 (60.8)
Prevalence of persistent biochemical hepatitis	52 (10.2)
Cause of biochemical hepatitis	
Viral hepatitis‡	
Reactivation of chronic HBV infection	5 (9.6)
Chronic HCV infection	7 (13.5)
Chronic HEV infection	5 (9.6)
CMV reactivation	8 (15.4)
Nonviral causes†	
Drug toxicity	7 (13.5)
Nonalcoholic fatty liver disease	3 (5.8)
Liver graft rejection	7 (13.5)
Biliary anastomotic stricture	5 (9.6)
Liver malignancies	2 (3.8)
Septic cholestasis	2 (3.8)
Recurrent pyogenic cholangitis	1 (1.9)

*CMV, cytomegalovirus; HBV, hepatitis B virus; HCV, hepatitis C virus; HEV, hepatitis E virus. †All results are no. (%) unless otherwise indicated. ‡All percentages based on no. patients with biochemical hepatitis.

### Patient History

A 56-year-old man underwent deceased-donor liver transplant on May 14, 2017, because of hepatocellular carcinoma complicating chronic HBV carriage. He received 1,000 mg hydrocortisone and 20 mg basiliximab (anti–interleukin-2 receptor mAb) as intraoperative antirejection prophylaxis and 4 units of platelets (derived from 4 separate blood donors) during the operation. His liver function tests (LFTs) reverted to normal, and he was discharged on posttransplant day 11. He was taking mycophenolate mofetil (500 mg 2×/d), tacrolimus (1 mg 2×/d), and prednisolone (5 mg 2×/d) as antirejection prophylaxis. He was also taking entecavir (0.5 mg 1×/d) for HBV suppression; serum HBsAg was negative 6 weeks after the transplant.

Routine phlebotomy on July 12 (day 59 posttransplant) revealed mild derangement of ALT to 74 U/L (reference 8–58 U/L). Other LFTs were normal. One week later, there was further derangement of parenchymal liver enzymes: ALT was 138 U/L, aspartate aminotransferase was elevated to 65 U/L (reference 15–38 U/L), γ-glutamyltransferase was 124 U/L (reference 11–62 U/L), and alkaline phosphatase was within reference limits at 70 U/L (reference 42–110 U/L). Complete blood count showed lymphopenia, at 0.88 × 10^9^ cells/L, although total leukocyte count was within reference levels.

The patient was empirically managed for acute graft rejection with increased immunosuppression using a 3-day course of methylprednisolone. Valganciclovir was prescribed for low-level whole blood CMV viremia of 5.31 × 10^2^ IU/mL. However, LFTs continued to deteriorate despite clearance of CMV viremia and increased immunosuppression. Liver biopsy showed nonspecific mild to moderate inflammatory infiltrate comprising small lymphocytes in the portal tracts. There were no viral inclusion bodies, and immunohistochemical staining for CMV and hepatitis B core antigens was negative. Results of testing for HBsAg in serum, HBV DNA in plasma, HCV antibody in serum, HAV IgM in serum, and HAV RNA in plasma and feces were all negative. HEV IgM was detected in serum collected on August 22 (day 100 posttransplant). Because of the serology result and ongoing LFT derangement, persistent HEV infection was suspected. A qRT-PCR targeting HEV-A was performed on patient fecal and plasma specimens; HEV-A RNA was not detected in either specimen. An RT-PCR capable of detecting all species within the *Orthohepevirus* genus detected amplicons (Technical Appendix Figure 1) in plasma, feces, and liver tissue. Sequencing confirmed that the products clustered with rat HEV-C strains.

### Viral RNA Kinetics and Effect of Ribavirin Therapy

**Figure 1:**
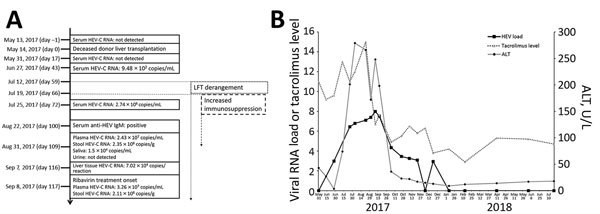
Natural course of HEV-C infection in a 56-year-old man at Queen Mary Hospital, Hong Kong. A) Timeline of major clinical events. All days are post transplant. B) Kinetics of liver function tests, tacrolimus levels (μg/L), and plasma HEV-C RNA load (log_10_ copies/mL) with relation to ribavirin therapy. ALT, alanine aminotransferase; HEV-C, *Orthohepevirus* *C*; LFT, liver function test.

The patient’s archived serum, saliva, urine, feces, and nonfixed liver tissue samples were retrieved for HEV-C RNA load testing using HEV-C qRT-PCR (Figure 1, panel A). Two pretransplant serum samples and 1 serum sample collected on day 17 after transplant did not contain HEV-C RNA. The first specimen with detectable HEV-C RNA was a serum sample collected 43 days after transplant, which contained an RNA load of 9.48 × 10^2^ copies/mL; this result preceded onset of LFT derangement by 3 weeks. After heightened immunosuppression in July and August, the HEV-C RNA load in blood steadily rose along with ALT (Figure 1, panel B). Variation in ALT correlated with the HEV-C RNA viral load by linear regression (R^2^ = 0.791). HEV-C RNA was also detected in feces, saliva, and liver tissue (Figure 1, panel A); feces contained the highest RNA load.

Immunosuppressant dosages were decreased after confirmation of HEV infection. However, ALT and HEV-C RNA loads continued to increase despite reduction of plasma tacrolimus levels by 55% and rebound of lymphocyte count to 2.27 × 10^9^ cells/L. Therefore, oral ribavirin 400 mg twice daily was started on September 7. ALT decreased within the first week after start of therapy and normalized within 1 month after starting ribavirin (Figure 1, panel B). HEV-C RNA loads also decreased to undetectable levels in plasma obtained on February 13, 2018. Ribavirin was stopped in April 2018, and HEV-C RNA in serum remained undetectable as of August 21, 2018, confirming sustained virologic response.

### Serologic Analysis

We retrospectively tested all available patient serum and plasma samples for HEV IgG and IgM ELISA using the Wantai ELISA kit. The patient’s serum before transplant was HEV IgG positive and IgM negative. HEV IgG and IgM optical density rose sharply from June 27, when HEV-C RNA was first detectable in blood, to July 25, when clinical hepatitis began (Technical Appendix Figure 2). Despite high IgG levels, HEV-C RNA continued to rise until ribavirin was started.

**Figure 2:**
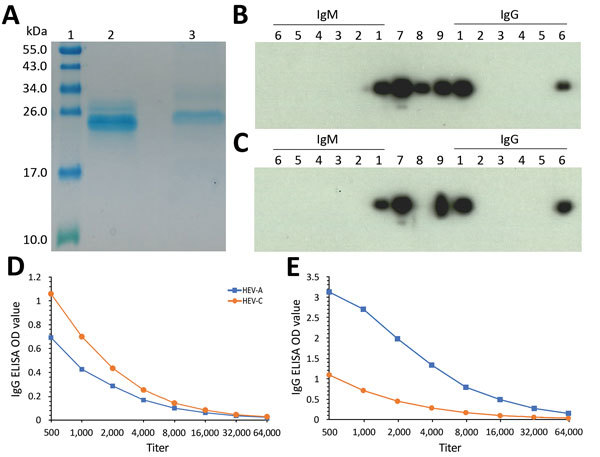
Serologic testing for HEV infection at Queen Mary Hospital, Hong Kong. A) Sodium dodecyl sulfate polyacrylamide gel electrophoresis gel showing purified HEV-A and HEV-C 239-aa recombinant proteins used in Western blot and ELISA. Lane 1, molecular weight marker; lane 2, HEV-A protein; lane 3, HEV-C protein. B–C) IgM and IgG Western blot using HEV-A protein (B) and HEV-C protein (C). Lane 1, patient serum (posttransplant day 100); lanes 2–5, individual platelet donor serum; lane 6, organ donor serum; lane 7, murine polyclonal serum against HEV-C; lane 8, specific monoclonal antibody against HEV-A; lane 9, cross-reactive monoclonal antibody against HEV-A and HEV-C. D, E) HEV-A and HEV-C ELISA IgG titers of patient pretransplant (D) and posttransplant serum (E) using an OD of 0.3 as assay cutoff as described in the Technical Appendix. HEV, hepatitis E virus; HEV-A, *Orthohepevirus* *A*; HEV-C, *Orthohepevirus* *C*; OD, optical density.

To characterize the serologic response, Western blot using purified HEV-A and HEV-C recombinant proteins (Figure 2, panel A) was performed. Two mAbs raised against HEV-A were used: 1 produced a band in HEV-A IgG blot but not in the HEV-C blot (lane 8; Figure 2, panels B, C) confirming specificity, and the other was cross-reactive against HEV-A and HEV-C (lane 9; Figure 2, panels B, C). Polyclonal serum raised in mice inoculated with HEV-C protein reacted in both blots, showing that the serum was cross-reactive (lane 7). Patient serum collected on day 100 after transplant (lane 1) was tested against HEV-A and HEV-C recombinant proteins. The serum specimen showed reactivity in both Western blots.

Two patient serum samples, 1 obtained 3 months before transplant and the other obtained on day 100 after transplant, were tested in IgG ELISAs using HEV-A and HEV-C protein-coated plates. The pretransplant serum (Figure 2, panel D) had cross-reactive antibodies against both HEV-A and HEV-C proteins (<2-fold difference in titer using OD cutoff of 0.3). However, the posttransplant serum (Figure 2, panel E) showed >16-fold rise in HEV-A IgG titer and markedly higher reactivity against HEV-A than against HEV-C (>4-fold difference in titer using a cutoff OD of 0.3).

### Liver Histologic and Immunohistochemical Analyses

**Figure 3:**
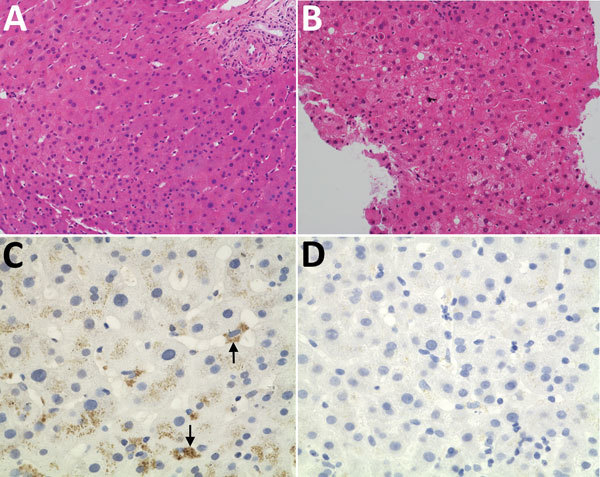
Histologic and immunohistochemical staining of liver tissue from a 56-year-old man at Queen Mary Hospital, Hong Kong. A, B) Liver tissue sections (original magnification ×200) stained with hematoxylin and eosin obtained at day 0 (A), showing normal hepatocyte architecture, and day 98 (B) after transplant showing progressive increase in hepatocyte ballooning and degenerative changes. C, D) Liver tissue section stained with cross-reactive monoclonal antibody (original magnification ×400); arrows show perinuclear antigen staining (C) and negative control with bovine serum albumin (D).

Serial liver biopsies showed progressively worsening hepatocyte ballooning and degenerative changes (Figure 3, panels A, B). Apoptotic hepatocytes were identified in the biopsy obtained on day 98 posttransplant (Figure 3, panel B). Immunohistochemical staining with the cross-reactive mAb showed positive perinuclear cytoplasmic signals (Figure 3, panel C), and negative control with bovine serum albumin instead of mAb showed no signals (Figure 3, panel D).

### Genomic Description

**Figure 4:**
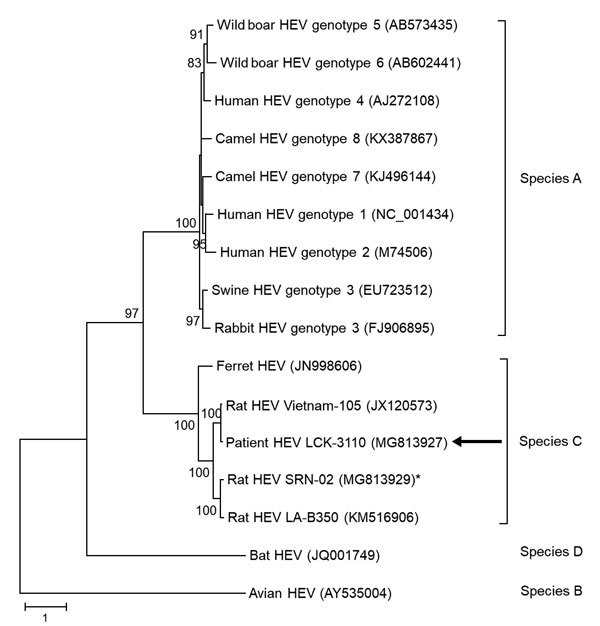
Phylogenetic analysis using complete open reading frame 2 nucleotide sequences of LCK-3110 and other hepatitis E virus strains. The tree was constructed using maximum-likelihood method with bootstrap values calculated from 1,000 trees. Only bootstrap values >70% are shown. GenBank accession numbers are provided. Arrows indicate strain LCK-3110; asterisk indicates strain SRN-02 detected in a street rodent in the epidemiologic investigation. Scale bar indicates mucleotide substitutions per site.

Complete genome sequencing of the patient’s fecal HEV isolate (LCK-3110) showed that the genome was 6,942 bp long (GenBank accession no. MG813927). Phylogenetic trees of the nucleotide and amino acid sequences of ORF1, ORF2, and ORF3 of HEV strains showed that LCK-3110 is most closely related to the Vietnam-105 strain (Figure 4; Technical Appendix Figure 3, panels A, B), sharing 93.7% nt identity. Because no phylogenetic incongruence was found on comparison of trees of the 3 genomic segments, recombination was unlikely (Table 2; Technical Appendix). To determine whether commonly used RT-PCRs for HEV nucleic acid amplification could detect HEV-C, we aligned published primer/probe sequences of HEV RT-PCRs (*19*–*22*) with complete genome sequences of HEV-A (genotype 1 reference strain) and HEV-C (strains LCK-3110, Vietnam-105, and LA-B350) using ClustalX 2.0 (http://www.clustal.org/clustal2/). Alignment revealed significant lack of homology with HEV-C at the 3′ end of either the forward or reverse primer for the assays described by Jothikumar et al. and Rolfe et al. (Technical Appendix Figure 4, panels A, B) (*20*,*21*). Our in-house HEV-A qRT-PCR is based on the primer/probe design of Jothikumar et al. and was unable to detect HEV-C in patient specimens (*20*). For the assays described by Mansuy et al. and Colson et al. (*19*,*22*), there was significant lack of matching of probe sequence (40%–45% mismatch) to HEV-C genomes (Technical Appendix Figure 4, panels C, D), which most likely would result in failure to detect any amplified nucleic acid.

**Table 2:** Comparison between nucleotide and deduced amino acid sequence identities of HEV strain LCK-3110 and other HEV strains

HEV strain (GenBank accession no.)	HEV species	Rat HEV strain LCK-3110							
Entire genome	Nucleotides, %				Amino acids, %		
	ORF1	ORF2	ORF3		ORF1	ORF2	ORF3
Genotype 1 (NC_001434)	HEV-A	57.6	56.4	60.9	55.0		50.0	56.3	31.0
Genotype 2 (M74506)	HEV-A	57.3	56.3	60.0	50.4		49.7	56.1	27.6
Genotype 3 (EU723512)	HEV-A	56.6	55.4	60.7	51.9		50.3	56.5	30.7
Genotype 4 (AJ272108)	HEV-A	56.5	55.4	59.8	55.4		49.7	56.4	31.0
Rabbit HEV (FJ906895)	HEV-A	56.0	54.9	59.9	51.6		50.1	56.4	27.6
Wild boar HEV (AB573435)	HEV-A	57.3	56.2	60.4	54.8		49.7	56.2	33.6
Wild boar HEV (AB602441)	HEV-A	56.8	55.7	59.6	54.5		50.3	56.5	31.9
Camel HEV (KJ496144)	HEV-A	55.9	54.9	59.4	53.5		50.5	56.2	32.2
Camel HEV (KX387867)	HEV-A	55.6	54.3	59.7	53.7		50.1	55.8	29.6
Rat HEV Vietnam-105 (JX120573)	HEV-C	93.7	93.3	95.2	96.4		98.2	98.0	95.1
Rat HEV LA-B350 (KM516906)	HEV-C	77.3	76.3	79.7	79.3		88.0	92.1	64.7
Ferret HEV (JN998606)	HEV-C	68.7	67.5	71.1	64.2		74.8	78.7	45.9
Bat HEV (JQ001749)	HEV-D	53.8	53.8	54.3	44.7		45.7	47.9	18.1
Avian HEV (AY535004)	HEV-B	53.5	54.0	53.0	46.4		45.6	43.5	24.8

*HEV, hepatitis E virus; ORF, open reading frame.

### Virus Culture

**Figure 5:**
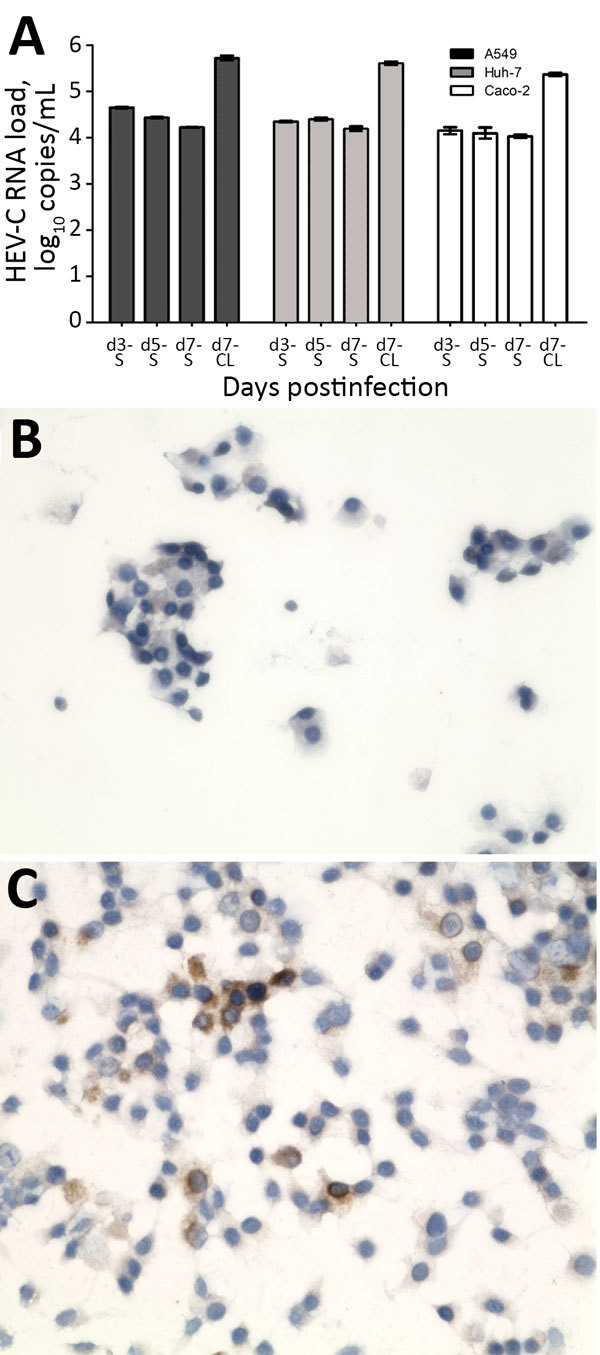
Isolation of HEV-C from 56-year-old male patient’s feces in cell culture, Queen Mary Hospital, Hong Kong. A) HEV-C RNA loads in culture S and day-7 CL of A549, Huh-7, and Caco-2 cell lines after inoculation by patient’s filtered fecal suspension. Mean of 3 replicates; error bars indicate SEM. B) Uninfected A549 cell monolayer stained with anti–HEV-C polyclonal antiserum. C) Infected A549 cell monolayer stained with anti–HEV-C polyclonal antiserum. Original magnification ×400. CL, cell lysate; HEV, hepatitis E virus; S, supernatant.

We detected HEV-C RNA in supernatants from all 3 cell lines (Figure 5, panel A) inoculated with patient’s feces at steady levels from day 3 to day 7 after inoculation. RNA loads in cell lysates were ≈1 log higher than concomitantly harvested supernatants, suggesting successful viral cell entry. Immunohistochemical staining (Figure 5, panels B, C) of A549 cell monolayers and immunofluorescence staining of infected Huh-7 and Caco-2 cells (Technical Appendix Figure 5) confirmed the presence of cytoplasmic HEV ORF2 antigen when stained with antiserum against HEV-C. These findings suggested abortive viral replication of HEV-C in human cell lines.

### Epidemiologic Investigation

The first clinical sample with detectable HEV-C RNA was obtained 43 days after transplant. HEV-C was not detected in serum samples obtained before transplant. Serum samples from the organ donor and all 4 platelet donors tested negative by IgM Western blot against HEV-C recombinant protein (Figure 2, panel C, lanes 2–6) and HEV-C qRT-PCR.

The patient’s house unit was located adjacent to a refuse chute. He had noticed rodent droppings but had never seen rats inside his home. A site visit to the housing estate was conducted on November 22, 2017. Rodent droppings were found around refuse collection bins on the ground floor and the floor where the patient lived. Twelve rodent fecal specimens, 2 swab samples from the drain, and 2 swab samples from the refuse room floor tested negative for HEV-C RNA. To expand the investigation, we retrieved archived rodent samples collected from the area around the patient’s housing estate (≈2.5-km radius) as part of preexisting pathogen surveillance programs. Spleen, kidney, liver, and rectal swab specimens from 27 rats were tested by qRT-PCR. The internal organs of 1 street rat (*Rattus norvegicus*) collected in 2012 tested positive for HEV-C RNA (strain name SRN-02). The ORF2 aa sequence of this isolate had 90.9% identity to LCK-3110.

## Discussion

Discovered in Germany in 2010, rat HEV variants have been detected in rodent samples in Asia, Europe, and North America (*23*–*26*). Because of high divergence from human-pathogenic HEV, rat HEV has been classified into a separate species, *Orthohepevirus* *C*, within the family *Hepeviridae* (*27*). The zoonotic potential of HEV-C is controversial. Virus-like protein ELISAs show possible subclinical infection among forestry workers in Germany and febrile inpatients in Vietnam, although interpretation of such studies is difficult because of serologic cross-reactivity between HEV-A and HEV-C (*15*,*28*). Immunocompetent rhesus macaques do not appear to be susceptible to experimental infection with a North America HEV-C isolate (*23*).

In this study, we detected HEV-C RNA in multiple specimens from a transplant recipient. The HEV-C infection manifested as persistent hepatitis, as shown by temporal correlation between blood HEV-C RNA detection and hepatitis onset, presence of HEV-C RNA in liver tissue, and normalization of liver function tests with viral clearance. These findings prove that HEV-C can infect humans to cause clinically significant illness and signal a need to reevaluate the importance of HEV-C as a human zoonosis among both immunocompromised and immunocompetent patients with hepatitis of unknown etiology.

The patient reported here acquired HEV-C infection despite having HEV IgG. Interpreted in parallel with the finding by Sanford et al. that inoculating pigs with HEV-C ORF2 protein did not protect them from HEV-A infection and low amino acid homology between HEV-A and HEV-C in critical immunogenic domains (*29*), our data suggest that HEV-A antibodies do not protect against HEV-C infection. The patient’s postinfection serum showed significantly higher reactivity in an HEV-A–specific ELISA than in an HEV-C ELISA; the humoral immune responses of persons with past HEV-A infection to de novo HEV-C infection are worthy of further study to identify whether anamnestic responses are mounted.

The patient’s HEV isolate had high nucleotide similarity to the HEV-C Vietnam-105 strain. It shared less homology with the North America LA-B350 strain, especially in the ORF3 domain, which is important for viral egress (*30*). Interspecies transmission could not be attributed to specific viral mutations. Future studies will need to include differences in zoonotic potential between HEV-C strains from Asia and the Americas.

The patient’s immunosuppression possibly enabled the virus to surmount the species barrier, as described previously for avian influenza (*31*,*32*). HEV-C infections may go undiagnosed because of amplification failure in RT-PCRs, which are designed based on HEV-A sequences (Technical Appendix Figure 3). The Wantai ELISA, based on HEV-A genotype 1, was able to detect IgM in this patient, but whether the assay is sensitive for HEV-C infection or was detecting only HEV-A–specific antibodies is uncertain. Therefore, we believe that specific RT-PCR is the most reliable method to diagnose HEV-C infections.

Our findings are also relevant to blood and organ donation safety. Because of the inability of commonly used RT-PCRs to detect HEV-C, transmission from asymptomatically infected immunocompetent donors may occur, even in countries that screen donated blood for HEV. Studies examining frequency of HEV-C contamination in blood products are needed to quantify this threat.

The patient lived in a housing estate with evidence of rat infestation in the refuse bins outside his home. We identified HEV-C in street rodents from the area, but the isolate was not closely related to the patient’s isolate. The route of transmission is unclear; we postulate that contamination of food by infected rat droppings in the food supply is possible. Other possibilities include reactivation of a subclinical infection in the patient posttransplant or a donor-derived infection from residual HEV-C in the transplanted organ. However, we found no serologic or virologic evidence of HEV-C infection in donor and recipient serum before transplant. An occult infection in the donated liver, which reactivated after transplant as described previously for HEV-A, cannot be completely excluded. Detailed studies are needed to ascertain the route of HEV-C infection in humans.

Technical Appendix Detailed methods for study of rat hepatitis E virus as cause of persistent hepatitis after liver transplant, Hong Kong, China.
