# Supplementary material for: Rat Hepatitis E Virus as Cause of Persistent Hepatitis after Liver Transplant
Source: Emerg Infect Dis. 2018 Dec;24(12):2241–50. doi: 10.3201/eid2412.180937 (PMC6256372; doi:10.3201/eid2412.180937)
Supplement: Technical Appendix — Detailed methods for study of rat hepatitis E virus as cause of persistent hepatitis after liver transplant, Hong Kong, China. [file 18-0937-Techapp-s1.pdf]

Article DOI: https://doi.org/10.3201/eid2412.180937

Rat Hepatitis E Virus as Cause of Persistent Hepatitis after Liver Transplantation

Technical Appendix

Detailed Study Methods

HEV Real-Time Reverse Transcription PCR (qRT-PCR)

200 μL of each sample was subjected to total nucleic acid extraction into 60 μL eluate using the EZ1 Virus Mini Kit v2.0 (Qiagen, Hilden, Germany). Primer & probe sequences, gene targets and product size of the 2 qRT-PCR assays specific for HEV-A and HEV-C are included in Technical Appendix Table 1. Quantitative real-time RT-PCR (qRT-PCR) assays were performed using QuantiNova Probe RT-PCR Kit (Qiagen) in a LightCycler 480 Real-Time PCR System (Roche, Basel, Switzerland). Each 20 μL-reaction mix contained 1× QuantiNova Probe RT-PCR Master Mix, 1× QN Probe RT-Mix, 0·8 μM forward and reverse primers, 0·2 μM probe and 5 μl template RNA. Reactions were incubated at 45°C for 10 min and 95°C for 5 min, followed by 50 cycles at 95°C for 5 s and 55°C for 30 s. Quantitation was achieved using plasmid standards prepared using the pCRII-TOPO vector (Invitrogen, Carlsbad, CA, USA) cloned with the target insert. Plasmid concentrations ranging from 10^2–10^6 copies/reaction were used to generate standard curves for each qRT-PCR run.

HEV Conventional RT-PCR

Conventional RT-PCR for HEV RNA detection in plasma, stool and liver biopsy samples was performed using primers listed in Technical Appendix Table 1. Reverse transcription for the pan-*Orthohepevirus* conventional RT-PCR assay was performed using the SuperScript III kit (Invitrogen). The reaction mixture (10 μL) contained RNA, first-strand buffer (50 mM Tris-HCl pH 8.3, 75 mM KCl, 3 mM MgCl2), 5 mM DTT, 50 ng random hexamers, 500 μM of each deoxynucleoside triphosphate (dNTP) and 100 U Superscript III reverse transcription. The mixtures were incubated at 25°C for 5 min, followed by 50°C for 60 min and 70°C for 15 min.

The PCR mixture (25 μL) contained cDNA, 1× PCR buffer II (10 mM Tris-HCl [pH 8.3], 50 mM KCl), 2 mM MgCl2, 200 μM of each dNTP, 1 μM forward and reverse primers and 1.0 U of Taq polymerase (Applied Biosystems, Foster City, CA, USA). PCR was performed using an automated thermocycler (Applied Biosystems) with a hot start at 95°C for 10 min, followed by 40 cycles of 94°C for 1 min, 55°C for 1 min, and 72°C for 1 min and a final extension at 72°C for 10 min. The PCR products were detected by agarose gel electrophoresis (staining gel with ethidium bromide, followed by visualization under UV light).

HEV Complete Genome Sequencing

PCR amplicon of the pan-*Orthohepevirus* RT-PCR assay was extracted using the QIAquick gel extraction kit (Qiagen). Both strands of the PCR product were sequenced twice with an ABI Prism 3130xl DNA Analyzer (Applied Biosystems) using primers listed in Technical Appendix Table 1. As the 235 bp RNA-dependent RNA polymerase (RdRp) sequences of HEV isolates obtained from patient specimens clustered with rat HEV-C strains, degenerate primers for complete genome amplification were designed by multiple alignment of the rat HEV-C genomes available in GenBank as per Technical Appendix Table 2. RNA extracted from the patient's stool (containing HEV isolate LCK-3110) was converted to cDNA by a combined random-priming and oligo(dT) priming strategy; this cDNA was used as the template for complete genome sequencing. The 5′ end of LCK-3110 was confirmed by rapid amplification of cDNA ends using Terminal Deoxynucleotidyl Transferase, recombinant (Invitrogen). Sequences were assembled and manually edited to produce final sequences of the viral genomes by BioEdit version 7.2.5 (NC State University, Raleigh, NC, USA).

HEV-A and HEV-C Peptide Expression

(5′-CATATGCTGTTAGGCGGCCTGCCAAC-3′ and 5′-CTCGAGCATCGGCACTGCAGCCGAG-3′) were used to amplify the gene encoding the 239-aa (aa) recombinant peptide corresponding to aa 368–606 of HEV-C ORF2. Primers (5′-CATATGATAGCATTGACCCTGTTTAATCT-3′ and 5′-CTCGAGAGCAGAGTGGGGTGCTAAAACAC-3′) were used to amplify the HEV-A gene encoding the recombinant peptide corresponding to aa 413–651 of HEV-A ORF2 (genotype 4). Amplified genes were cloned into the *Nde I* and *Xol I* sites of bacterial expression vector pETH in frame and downstream of the series of 6 histidine residues. The recombinant 239 aa HEV-A

and HEV-C proteins were expressed in *Escherichia coli* and purified by using the Ni-nitrilotriacetic acid affinity chromatography assay (Qiagen) according to manufacturer instructions. The HEV-A and HEV-C proteins had a 219 aa overlapping fragment and shared 52% sequence homology. The sequence alignment of the 2 proteins is as follows:

```
HEV-A       IALTLFNLADTLLGGLPTELISSAGGQLFYSRPVVSANGEPTVKLYTSVENAQQDKGIAI 60

HEV-C      ———-LLGGLPTDLVSNAGGQLFYGRPQVSENGEPSVKLYTSVEAAQLDHGVTI        49

                       *******:*:*.*******.** ** ****:******** ** *:*::*


HEV-A       PHDIDLGESRVVIQDYDNQHEQDRPTPSPAPSRPFSVLRANDVLWLSLTAAEYDQTTYGS 120

HEV-C       PHDIDLGVSAITLQDFDNQHLQDRPTPSPAPARPITNWRSGDVVWVTLPSAEYAQSQSAM 109

            ******* *:.:**:**** **********:**::  *:.**:*::*:*** *:   .


HEV-A       STNPMYVSDTVTFVNVATGAQGVARSLDWSKVTLDGRPLTTIQQYSKTFFVLPLRGKLSF 180

HEV-C       GSHPAYWSEEATIINVATGQRAAVSSIKWDQVTLNGKALHKETHSGLVYYQLPLMGKINF 169

            .::* * *: .*::*****:... *:.*.:***:*: * . : . .:: *** **:.*


HEV-A       WEAGTTKAGYPYNYDTTASDQILIENAAG-HRVCISTYTTNLGSGPVSISAVGVLAPHS  238

HEV-C       WQQGTTKAGYTYNYNTTDSDSLWVWWDGGSKAYLYISTYTTMLGAGPVNITGLGAVGPNP 229

            *: ******* ***:** **.::   .*   : ****** **:***.*:.:*.:.*:


HEV-A       A——                                                          239

HEV-C       VDQASAAVPM                                                   239
```

Antibodies Against HEV-A and HEV-C

Thirty micrograms of purified recombinant HEV-C protein mixed with an equal volume of complete Freund's adjuvant (Sigma-Aldrich, St. Louis, MO, USA) was injected subcutaneously into mice followed by 4 injections of incomplete Freund's adjuvant (Sigma-Aldrich) at 14-day intervals. Polyclonal antiserum collected after the fourth injection were used for serologic and immunohistochemical assays.

HEV In-House Serologic Assays

For the Western blot assay, purified recombinant HEV-A and HEV-C proteins were separated electrophoretically in a 12% gel and transferred to a nitrocellulose membrane. Western blot was performed in a Mini-PROTEAN II Multiscreen Apparatus (Bio-Rad, Hercules, CA, USA). The membrane was incubated with polyclonal anti-serum (dilution of 1:10,000) from mice immunized with purified HEV-A and HEV-C proteins, monoclonal antibodies (1 μg/mL) against HEV-A, patient and organ/blood donor serum (in dilution of 1:500) for 1 h at 37°C. After washing, the membrane was incubated with horseradish peroxidase (HRP; Sigma-Aldrich) conjugated goat anti-human (IgG or IgM) and goat anti-mouse antibodies for 30 min at 37°C, and developed by incubation with Advansta ECL WesternBright Quantum Detection Kit (Advansta, Menlo Park, CA, USA).

For the HEV-A and HEV-C in-house ELISAs, 96-well microwell plates (Costar, Corning, NY, USA) were coated with purified recombinant HEV-A and HEV-C proteins. 100 μL of serum from three asymptomatic blood donors who tested negative in the Wantai HEV IgG ELISA and both HEV-A and HEV-C IgG Western blots was diluted 1:500 in 0.1% bovine serum albumin (BSA). Diluted serum was added to the ELISA plates (2 replicates) and incubated at 37°C for 1 h. After a washing step, goat anti-human-horse radish peroxidase (100 μL/ well) was added to plates followed by incubation at 37°C for 30 min. After washing, tetramethylbenzidate substrate was added. The reaction was stopped after 10 min by addition of 0.3 N sulphuric acid. Plates were examined in an ELISA plate reader at 450 nm. The mean optical density (OD) of these negative control sera was 0.152 (range 0·132–0·171; SD ± 0.017) for HEV-C ELISA and 0.178 (range 0.169–0.187; SD ± 0·008). Based on these findings, a tentative ELISA cutoff of 0.3 was designated. As subsequent ELISA experiments were quantal measurements using serial dilutions of patient serum, a precise cutoff is not necessary. For the ELISA experiment, patient serum was serially diluted 2-fold from 1:500 to 1:64,000 in BSA followed by HEV-A and HEV-C IgG ELISA as described above. The OD values were plotted against serum dilution. The IgG titer of a serum specimen was defined as the reciprocal of the highest dilution that gave an OD value above the cutoff. Using the tentative cutoff of 0.3, a serum sample would be considered to be specifically reactive against a particular HEV antigen (i.e., HEV-A or HEV-C) if there was a 4-fold difference in IgG titers between the 2 ELISAs. For example, if the IgG titer above the

cutoff line was 4,000 by HEV-A ELISA and 8,000 by HEV-C ELISA, the serum would not be considered to be more reactive against 1 HEV antigen over the other. Conversely, if the HEV-A ELISA IgG titer was 4,000 and HEV-C ELISA IgG titer was 16,000, then the serum would be considered to be more reactive against HEV-C than HEV-A.

Cell Culture

The patient’s stool was diluted in phosphate buffered saline (PBS) and filtered to produce a 10% suspension. The suspension was further diluted 1:10 (for A549 and Huh-7) and 1:100 (for Caco-2) and 300 μL of suspension was used to inoculate cells in 12-well plates at an estimated multiplicity-of-infection of 5 HEV genome equivalents/ cell. Inoculum was removed after 1 h and replaced with minimum essential medium supplemented with 1% fetal calf serum. Cell lines were incubated at 37°C and were examined for cytopathic effect (CPE) daily. On day 3 and day 5, 250 μL of supernatant was collected and replenished with fresh medium. Cell monolayers were maintained for 7 d before harvesting of cell lysate. A549 cell monolayers were inoculated in chamber wells for immunohistochemical staining.

Immunohistochemical and Immunofluorescence Staining

Immunofluorescence staining of infected and uninfected A549, Huh-7, and Caco-2 7-day cell lysates was performed using anti-HEV-C polyclonal serum and anti-HEV-A monoclonal antibody. Briefly, infected and uninfected cells (as a negative control) were washed twice with PBS and fixed on slides in cold acetone at –20°C for 10 min. Monolayers were then inoculated with mouse serum (diluted 1:100) for 1 h at 37°C. After washing 3 times with PBS, cells were incubated with FITC-conjugated goat anti-mouse IgG antibody (Invitrogen) (1:40 dilution) for 30 min. After washing 3 times in PBS, the cells were counter-stained with 0.25% Evans Blue for 15 min. After a final washing step and addition of mounting fluid, stained cells were visualized using a fluorescence microscope. Cells showing apple-green fluorescence in the cytoplasm were considered to be HEV infected.

De-paraffinized liver tissue sections were treated with antigen unmasking buffer (Vector Laboratories, Burlingame, CA, USA) and hydrogen peroxide block. Slides were then incubated with primary reagent (either cross-reactive monoclonal antibody or bovine serum albumin) overnight at 4°C. After rinsing, slides were incubated with biotin conjugated goat anti-mouse IgG at room temperature for 30 min. Rinsed slides were then incubated with HRP-streptavidin

followed by color development using 3, 3′-diaminobenzidine (Vector Laboratories). Liver tissue section slides were counterstained with Gill’s hematoxylin and examined using light microscopy.

Description of LCK-3110 Complete Genome

Predicted genomic organization of LCK-3110 was similar to other rat HEV isolates: from 5′ to 3′ ends, it consists of a 5′-untranslated region at nucleotide positions 1–10, ORF1 at nt 11–4903, ORF3 at nt 4920–5228 overlapping with ORF2 at nt 4931–6865 and 3′ UTR at nt 6866–6942 (including poly-A tail). An additional putative ORF, corresponding to ORF4 (nt 27–578) in the Vietnam-105 rat HEV-C strain genome (GenBank accession no. JX120573), was also found in LCK-3110. LCK-3110 is most closely related to the Vietnam-105 strain, sharing a nucleotide identity of 93.7%. No phylogenetic incongruence was found on comparison of trees of the 3 genomic segments, therefore recombination was unlikely.

Alignment of the 151-aa segment corresponding to the highly immunogenic E2s domain of HEV-A ORF2 (aa 455–aa 603 of HEV-A genotype 1 sequence) showed 98.6% homology between the LCK-3110 and Vietnam-105 strains. On the other hand, homology between LCK-3110 and HEV-A genotype 1 (Xinjiang strain; GenBank accession no. NC001434) E2s domain was only 47.7%, which was even lower than the overall ORF2 sequence amino acid homology. In-silico epitope analysis of the target residue sites of the 8C11 HEV-A neutralizing monoclonal antibody showed that only 1 out of 6 residues were conserved between LCK-3110 and the Xinjiang strain with a serine to glutamic acid substitution at the critical host-specificity defining residue aa 497 (HEV-A genotype 1 numbering).

Technical Appendix Table 1. Primers and probes targeting hepatitis E*

HEV species targeted (gene target)		Primer/probe sequence (5′→3′)	Product size, bp	PCR methodology
Pan-*Orthohepevirus* (RdRp)	Forward	ATGGTAAAGTGGGNCARGGNAT	235	Conventional PCR
	Reverse	CCAAGCGAGAAATTRTTYTGNGT		
Orthohepevirus A (ORF2)	Forward	GGTGGTTTCTGGGGTGAC	70	qRT-PCR
	Reverse	AGGGGTTGGTTGGATGAA		
	Probe	FAM- TGATTCTCAGCCCTTCGC -BHQ1		
Orthohepevirus C (ORF1)	Forward	CTTGTTGAGCTYTTCTCCCCT	69	qRT-PCR
	Reverse	CTGTACCGGATGCGACCAA		
	Probe	HEX- TGCAGCTTGTCTTTGARCCC -IABkFQ		

*ORF, open reading frame; qRT-PCR; quantitative real-time reverse transcription PCR; RdRp: RNA-dependent RNA polymerase.

Technical Appendix Table 2. Primers used for complete genome sequencing of LCK-3110*

Primer name	Sequence (5′ - 3′)	Position
LPW418	GACCACGCGTATCGATGTCGACTTTTTTTTTTTTTTTTTV	1st round PCR for 5′ and 3′ ends
ratHEV-5RACE-R1	GTGAATGACATTGGCGTGCT	303–322 (1st round)
LPW417	GACCACGCGTATCGATGTCGAC	nested PCR for 5′ and 3′ ends
ratHEV-5RACE-R2	CGGATGCGACCAAGAAACAG	189–208 (nested)

Primer name	Sequence (5′ - 3′)	Position
ratHEV-1F	CGATGGAGACCCATCAGTATGT	9–30
ratHEV-1R	GCGTATARAGTGTGCATNCCATG	551–573
ratHEV-16F	ACATCCGCCGTTGCATTCT	396–414
ratHEV-16R	ACTCTGGTCGCCTTAATCCA	725–744
ratHEV-2F	GATCTACATCCGCGTGAGGT	512–531
ratHEV-2R	TAAAAMCCTGCYGAAACCCA	1340–1359
ratHEV-3F	CACGCAGGGYATATCWATGGG	1183–1203
ratHEV-3Rm	ATGAAACAACCGCACACCTG	1466–1485
ratHEV-10F	TGGGTTTCRGCAGGKTTTTA	1340–1359
ratHEV-10R	ATYGGGCCMGGTGTCCAAGA	1856–1875
ratHEV-18F	CGGTATGAAGTTGGCAGGCT	1642–1661
ratHEV-18R	CGTGTATTATCGGCCTGGGT	2260–2279
ratHEV-4F	GATGGBGCAGCNGTTTATGA	2075–2094
ratHEV-4Rm	AACCARGCYTGCATGGACTC	2411–2430
ratHEV-24F	GATTATAGGGTCCGCCAAGA	2291–2310
ratHEV-26R	GGTACAATAACCAGGTCACAGTC	2798–2820
ratHEV-5F	AAGTTTRTTGGYAATGCCCA	2699–2718
ratHEV-19R	GTGGTAGACATCCCTGGGTA	3131–3150
ratHEV-11F	CTAGARCCCACRGARTGGCG	3059–3078
ratHEV-11R	GTKATCGANCCAGGGTGCAT	3230–3249
ratHEV-20F	GATGTTCACCGGGGACAGT	3172–3190
ratHEV-20R	TTGGCGACTATGGCCTTCTC	4097–4116
LPW7308 (F)	ATGGTAAAGTGGGNCARGGNAT	4020–4041
LPW7311 (R)	CCAAGCGAGAAATTRTTYTGNGT	4232–4254
ratHEV-12F	GCGTGCAGAGTGTTTGAAAATGA	4190–4212
ratHEV-12R	GAACAGCAAAAGCACGAGCA	4947–4966
ratHEV-13Fm	GCGGATGGAACTCAAGGAAT	4843–4862
ratHEV-13R	ATTGGCGACTGCCCGGCATC	5207–5226
ratHEV-21F	GCTATCACCAACCAACCCTT	5095–5114
ratHEV-21R	GGTGGACGTGATGGAGTTC	5569–5587
ratHEV-14F	CGACTGAGGCYTCNAATTATGC	5424–5445
ratHEV-14R	TGCACRTCCTGCATRAACC	5940–5958
ratHEV-22F	ACACCCGGTAATACCAACAC	5831–5850
ratHEV-22R	ATAGTCGCCTCCTCGGACCA	6377–6396
ratHEV-15F	TCCGGYGATGTKGTGTGGGT	6296–6315
ratHEV-15R	ACTCGCGCMATAGCWTCAGC	6809–6828
ratHEV-23F1	TTTGTGGGTGTGGTGGGATG	6601–6620 (1st round)
ratHEV-23F2	GGGCTCTAAGGCTTATCTCTAT	6622–6643 (nested)

*Primer position corresponding to the genome sequence of HEV strain LCK-3110. Primers were derived from the following HEV-C sequences in GenBank: Vietnam-105 (GenBank accession no. JX120573), ratELOMB-131 (GenBank accession no. LC145325), ratIDE079F (GenBank accession no. AB847305), rat/R63/DEU/2009 (GenBank accession no. GU345042), LA-B350 (GenBank accession no. KM516906), and rat/Mu09/0685/DEU/2010 (GenBank accession no. JN167537). F, forward; HEV, hepatitis E virus; R, reverse.

Technical Appendix Table 3. Primers used for sequencing of open reading frame 2 of street rodent isolate SRN-02*

Rat HEV	Primer	Sequence 5′→3′
SRN-02	HEV-8F	GACTCTGACHGTCCCTCAGTC
	HEV-8R	GTAATGTVACCCACACMACATC
	HEV-9F	GCTGTSAGGTTYATGCAGGA
	LPW418	GACCACGCGTATCGATGTCGACTTTTTTTTTTTTTTTTV
	LPW417	GACCACGCGTATCGATGTCGAC
	HEV-7F	TGGAACACNGTCTGGAAYATGGC
	HEV-12R	GAACAGCAAAAGCACGAGCA
	LPW36773	GCCGTATGGAGCTGAAGGA
	LPW36725	CGCATACCCACCCACCGAGT
	LPW36726	TGTGGGTCTGGTGGGATGG

*HEV, hepatitis E virus.

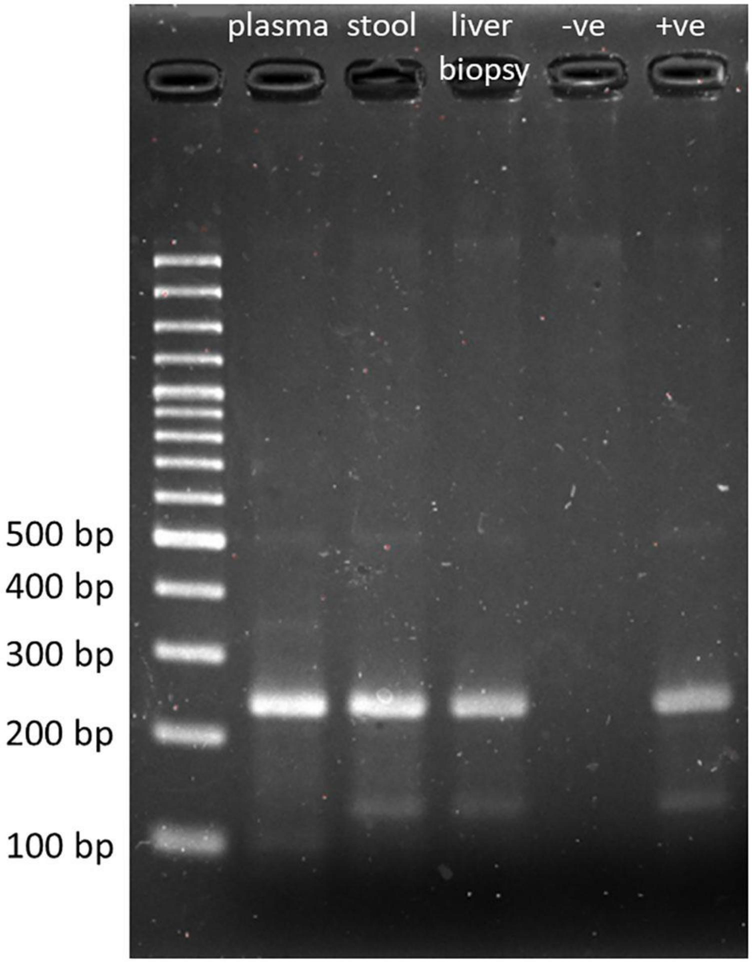

Technical Appendix Figure 1. Detection of hepatitis E virus–C RNA in patient clinical specimens. Gel photograph showing a 235-bp PCR product in patient plasma, stool, and liver tissue after amplification using pan-*Orthohepevirus* primers. –ve: negative control. +ve: positive control

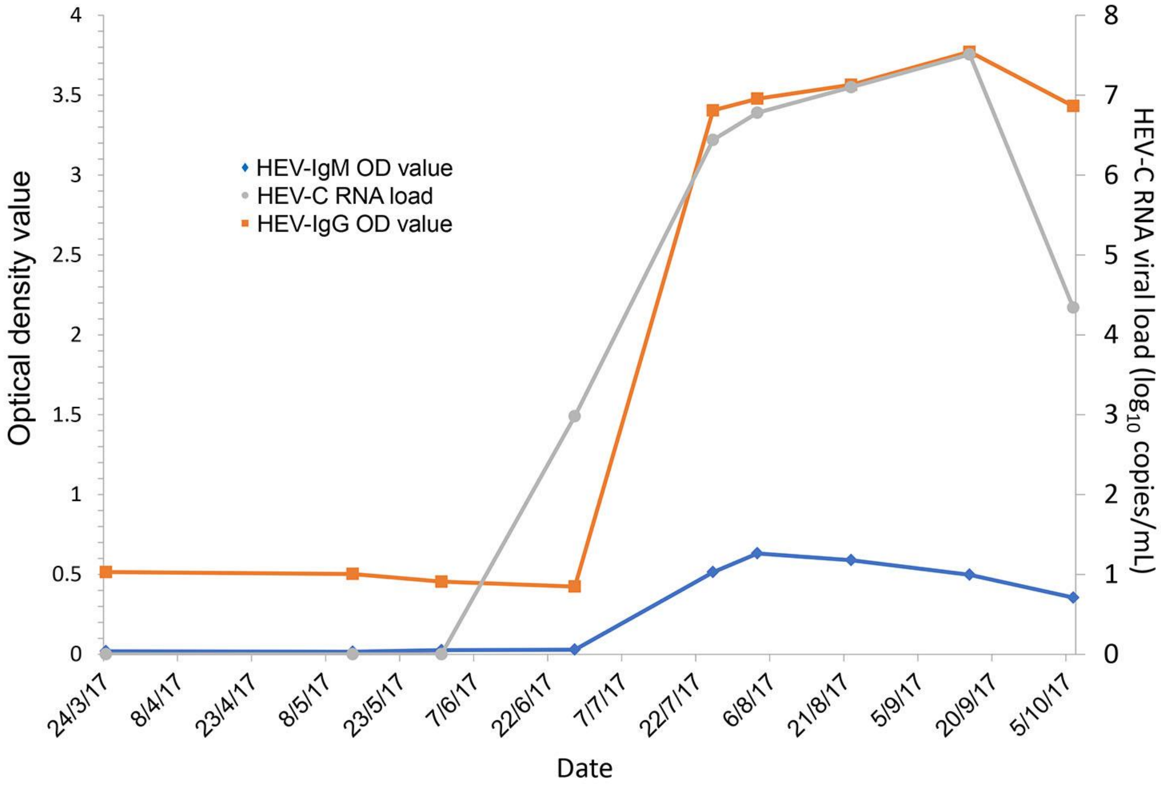

Technical Appendix Figure 2. Wantai HEV IgM and IgG OD values and HEV-C blood RNA loads over time. HEV-C RNA loads in peripheral blood continuously rose despite significant production of HEV IgG as measured by the commercially available Wantai HEV IgG ELISA kit, which uses antigens based on HEV-A. HEV, hepatitis E virus; OD, optical density.

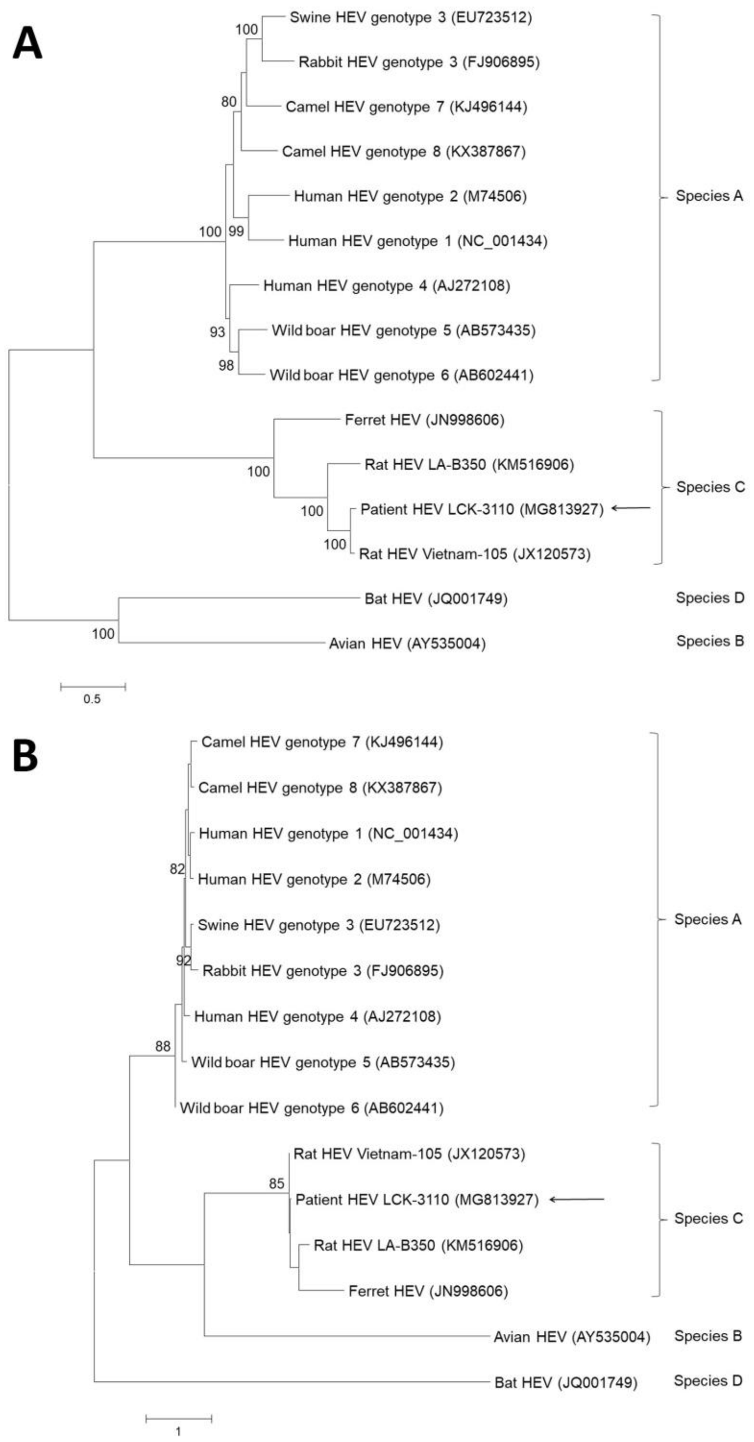

Technical Appendix Figure 3. Phylogenetic analysis using complete (A) open reading frame (ORF) 1 and (B) ORF3 nucleotide sequences of LCK-3110 and other HEV strains. The trees were constructed using maximum-likelihood method with the model GTR+G, with bootstrap values calculated from 1,000 trees. Only bootstrap values >70% are shown. Arrows indicate the strain LCK-3110. HEV, hepatitis E virus. Scale bar indicates nucleotide substitutions per site.

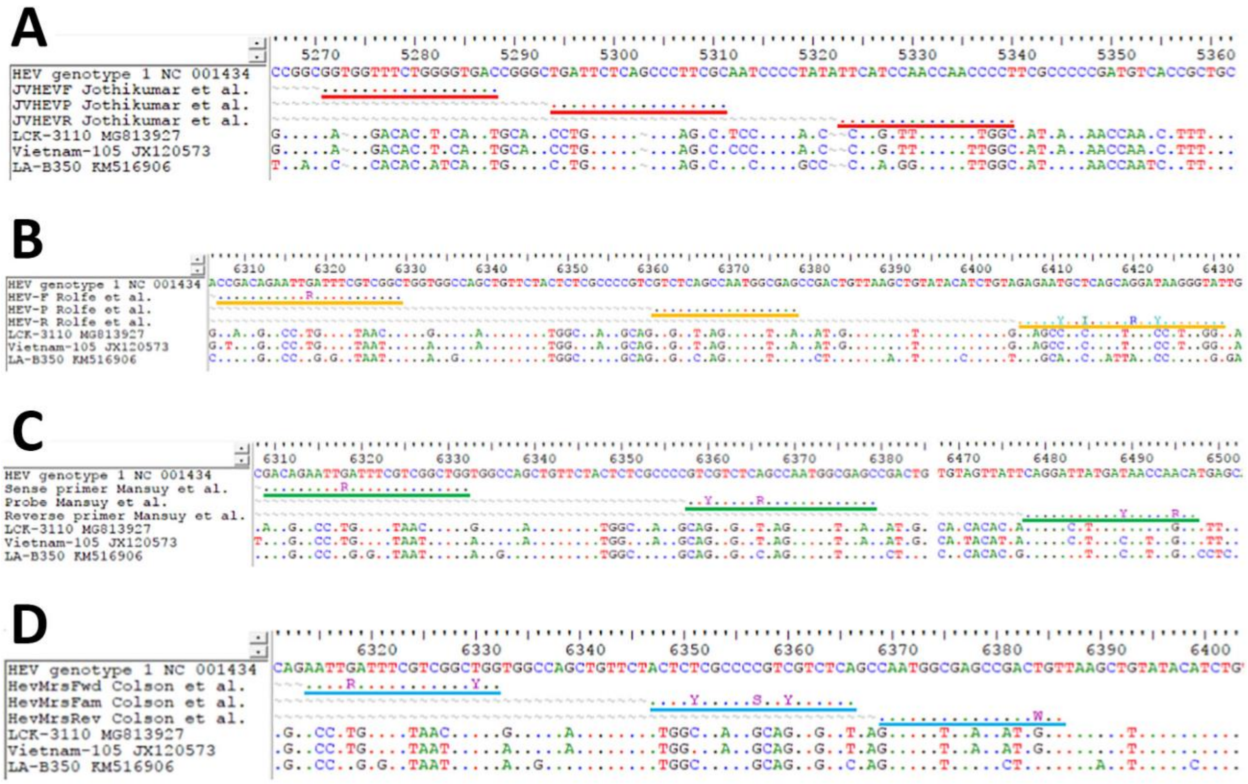

Technical Appendix Figure 4. Multiple sequence alignment of open reading frame (ORF) 2 of HEV genotype 1 (HEV-A), rat HEV (HEV-C) and primer and probe sequences (for HEV-A detection). Primer and probe sequences were adopted from (A) Jothikumar et al., (B) Rolfe et al., (C) Mansuy et al. and (D) Colson et al. HEV, hepatitis E virus.

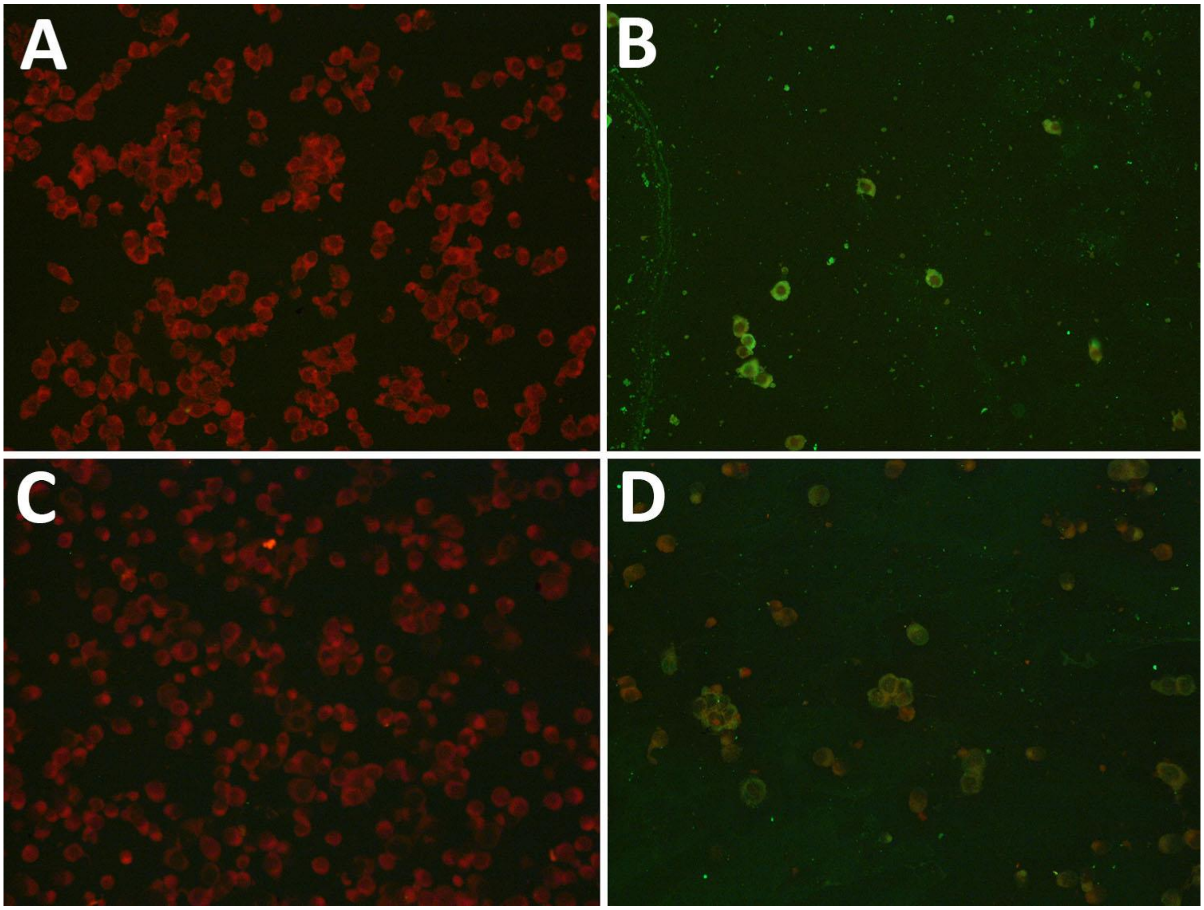

Technical Appendix Figure 5. Immunofluorescence staining of cell culture lysates. Uninoculated permeabilized Huh-7 cells (A) were stained with anti–hepatitis E virus–C (anti–HEV-C) polyclonal antiserum; Huh-7 cells harvested after 7 days of inoculation with patient's stool filtrate were stained with anti-HEV-C polyclonal antiserum (B). Uninoculated permeabilized Caco-2 cells (C) were stained with anti-HEV-C polyclonal antiserum; Caco-2 cells harvested after 7 days of inoculation with patient's stool filtrate were stained with anti-HEV-C polyclonal antiserum.
